# Annual acknowledgement of manuscript reviewers

**DOI:** 10.1186/s13584-016-0067-6

**Published:** 2016-03-10

**Authors:** Bruce Rosen, Avi Israeli

**Affiliations:** Israel Journal of Health Policy Research (IJHPR), Myers-JDC-Brookdale Institute, Jerusalem, Israel; Israel Journal of Health Policy Research (IJHPR), Hebrew University - Hadassah Medical Center, Jerusalem, Israel

## Abstract

The Editors of *Israel Journal of Health Policy Research* would like to thank all reviewers, both external and Editorial Board Members, who have contributed to the journal in 2015, and whose valuable support continues to be essential to the success of the journal.

**David Abrams**

USA

**Bruria Adini**

Israel

**Arnon Afek**

Israel

**Moty Amar**

Israel

**Eric Amster**

Israel

**Emilia Anis**

Israel

**Paul Appelbaum**

USA

**Jonathan Arbelle**

Israel

**Nachman Ash**

Israel

**Shai Ashkenazi**

Israel

**Toni Ashton**

New Zealand

**Alexander Aviram**

Israel

**Judit Bar-Ilan**

Israel

**Orna Baron-Epel**

Israel

**Yehuda Baruch**

Israel

**Eliezer Be'eri**

Israel

**Robert Bellmaker**

Israel

**Jochanan Benbassat**

Israel

**Gabi Bin Nun**

Israel

**Marvin Birnbaum**

USA

**Haim Bitterman**

Israel

**Malke Borow**

Israel

**Shuli Brammli-Greenberg**

Israel

**Danny Brom**

Israel

**Ronit Calderon-Margalit**

Israel

**David Chinitz**

Israel

**Gabriel Chodick**

Israel

**Sarah Clark**

USA

**Eyal Cohen**

USA

**Arnon Dov Cohen**

Israel

**Marc Cohen**

United Arab Emirates

**Nihaya Daoud**

Israel

**Ehud Davidson**

Israel

**Raz Dekel**

Israel

**Sarah Dine**

USA

**Francois Dionne**

Canada

**Milka Donchin**

Israel

**Don Downing**

USA

**Erin Dupree**

USA

**Arthur Eidelman**

Israel

**Eldad Elnekave**

Israel

**Linda Emanuel**

USA

**Ronit Endevelt**

Israel

**Leon Epstein**

Israel

**Dani Filc**

Israel

**Diana Flescher**

Israel

**Gary Freed**

USA

**David Garr**

USA

**Saralee Glasser**

Israel

**Shimon Glick**

Israel

**Sherry Glied**

USA

**Avishay Goldberg**

Israel

**Dan Greenberg**

Israel

**Pedro Greer Jr**

USA

**Itamar Grotto**

Israel

**Nurit Guttman**

Israel

**Jack Hadley**

USA

**Jonathan Halevy**

Israel

**James Halperin**

USA

**Paul Hansen**

New Zealand

**Yafa Haron**

Israel

**Anthony Heymann**

Israel

**Rosemary Hiscock**

UK

**Ohad Hochman**

Israel

**Dominic Hodgkin**

USA

**Marcia Javitt**

USA

**Nir Kaidar**

Israel

**Eddy Karnieli**

Israel

**Orit Karnieli-Miller**

Israel

**David Katz**

Israel

**Galit Kaufman**

Israel

**Jean Kervasdoue**

France

**Yan Kim**

USA

**Shmuel Klang**

Israel

**Ehud Kokia**

Israel

**Richard Kravitz**

USA

**Mooli Lahad**

Israel

**Bruce Landon**

USA

**Luís Lapão**

Portugal

**Leonard Leibovici**

Israel

**Eran Leitersdorf**

Israel

**Debra Lerner**

USA

**Moshe Leshno**

Israel

**Hagai Levine**

Israel

**Daphna Levinson**

Israel

**Itzchak Levy**

Israel

**Yoram Maaravi**

Israel

**Orly Manor**

Israel

**Carmi Margolis**

Israel

**Talya Miron-Shatz**

Israel

**Brian Morris**

Australia

**Yehuda Neumark**

Israel

**Marc Nivet**

USA

**Joseph Pliskin**

Israel

**Lion Poles**

Israel

**Rosalind Raine**

UK

**Nosaiba Rayan**

Israel

**Shmuel Reis**

Israel

**Ronli Rotem**

Israel

**Alvin Roth**

USA

**Siegal Sadetzki**

Israel

**Richard Saltman**

USA

**Stephen Schoenbaum**

USA

**Sigal Shafran -Tikva**

Israel

**Varda Shalev**

Israel

**Jeffrey Shames**

Israel

**Ira Sharkansky**

Israel

**Yaniv Sherer**

Israel

**Fiona Sim**

UK

**Joann Sperl-Hillen**

USA

**Shelley Sternberg**

Israel

**Aviad Tur-Sinai**

Israel

**Baruch Velan**

Israel

**Tim Watson**

UK

**Michael Weingarten**

Israel

**Shimon Weitzman**

Israel

**Rachel Wilf Miron**

Israel

**Yael Yishai**

Israel

**Deena Zimmerman**

Israel

